# Upright Open MRI (MRO) Evaluation of the Anatomic Effects of Yoga Postures on the Bladder Neck and Urethra

**DOI:** 10.3390/diagnostics15060723

**Published:** 2025-03-13

**Authors:** Andrew Macnab, Lynn Stothers

**Affiliations:** 1 Departments of Urology and Pediatrics, University of British Columbia, 4480 Oak Street, Vancouver, BC V6H 3V4, Canada; 2 Stellenbosch Institute for Advanced Study, Wallenberg Research Centre, Stellenbosch 7600, South Africa; 3 Division of Urogynecology and Reconstructive Pelvic Surgery, Department of Urology, University of California, 200 UCLA, Medical Plaza #140, Los Angeles, CA 90095, USA; mstothers@mednet.ucla.edu

**Keywords:** bladder neck descent, functional anatomy, lower urinary tract symptoms (LUTS), stress urinary incontinence, urethral mobility

## Abstract

**Background/Objectives**: Upright open magnetic resonance imaging allows the impact of posture and gravity to be evaluated. Randomized controlled trials of yoga for treating urinary incontinence (UI) in women show significant clinical benefit, yet the anatomic impact of this therapy on the lower urinary tract remains unelucidated. This study tested the hypothesis that open MRI scans can be obtained with sufficient detail to visualize the bladder neck and urethra. **Methods**: We scanned a volunteer subject using a 0.5 Tesla MRO Open Evo scanner to obtain axial and sagittal T2-weighted pelvic scans during poses used in yoga therapy. To obtain images with the necessary detail, we employed variations in sequencing during scanning of each individual pose. The changes observed in the bladder neck and urethral outline in each pose were then compared to baseline supine images. **Results**: Images with sufficient anatomic detail were obtained in each of the four poses studied. These scans identified that the urethral outline changes anatomically based on the posture adopted and is dynamic with regional alternations evident in caliber during specific yoga poses. **Conclusions**: Open MRI can identify anatomical changes involving the bladder neck and urethra that occur during yoga poses used in the treatment of UI in women; these likely relate to effects of posture and gravity. Open MRI offers a way to elucidate the anatomic effects that specific yoga poses generate and to identify those with the potential to be most beneficial clinically to women as a form of therapy.

## 1. Introduction

The configuration of upright open MRI scanners with a vertical gap in the magnet allows patients to be imaged in positions where the effects of posture and gravity affect functional anatomy in the body. The ability to scan patients in several different weight-bearing positions is recognized to aid demonstration of occult pathology not visualized in supine imaging [1]. This ability has been predominantly used for the investigation of orthopedic conditions but has also been shown to have value in women with urinary incontinence (UI) due to pelvic floor pathology [2,3,4]. A significant precipitating factor leading to UI in women is the stress gravity exerts on the pelvic organs when an upright posture is adopted.

UI, involuntary loss of urine, is a major clinical problem which affects millions of women worldwide; in this paper the terms ‘woman’ and ‘women’ are used as they were in the references cited. The incidence of UI increases with advancing age, and estimates are that up to 15% of middle-aged women are affected [5], with 32% of women over 80 reporting symptoms [6]. The most common form of UI is stress urinary incontinence (SUI) where physical activity precipitates symptoms; there are also urge-related and mixed forms [7].

UI is one of a growing number of chronic health conditions in which yoga has been studied as a form of therapy, either as a conservative intervention or as an adjunct to pharmacologic management, and has been reported to provide symptomatic benefit both as a primary measure and as an adjunct to more invasive treatments [5,8,9,10]. However, it is not currently clear how symptom relief is achieved; many factors could potentially contribute to the relief of symptoms accrued and the reasons why women with UI benefit from yoga have yet to be elucidated [7]. Specifically, the anatomic changes that logically could underlie the positive effects of yoga as therapy have yet to be identified [5,11,12]. Immediate temporary effects may include lifting and or closing the bladder neck, and long-term effects may generate sphincter hypertrophy, an improvement in the pubococcygeal angle or enhance pelvic floor muscle function thus restoring functional integrity that alleviates symptoms.

It has been proposed that the inclusion of particular poses (asanas) may be integral to yoga regimens that effectively treat UI; specific yoga poses believed to be helpful and that have been tested include the chair pose (Utkatasana), triangle pose (Trikonasana), and squat pose (Malasana) [5]. In a clinical context, because yoga is considered safe and easy to perform, it is a benign intervention for treating UI that could potentially benefit millions of women [13,14]. However, while it is the reduction in symptoms that is important for women with UI, the literature calls for identification of which yoga poses achieve an anatomic effect because these poses are seen as the ones most likely to help women with UI achieve symptomatic relief. This presumption assumes that positive functional changes in structures influencing continence are associated with effects on their position, strength, caliber, length or thickness, and that these changes translate into functional improvement and symptom relief; hence, once identified, specific poses deemed to generate potentially beneficial effects would be incorporated into therapeutic yoga regimens and further studied [5,11,12].

In this context, studies to date include clinical trials examining the ability of yoga to enhance clinical pelvic floor rehabilitation and generate symptomatic improvement for lower urinary tract symptoms (LUTS). These are summarized in a scoping review of eight studies [15] and a Cochrane review that evaluated two randomized controlled clinical trials [5]. One potential mechanism identified was that relative activation of the pelvic floor muscles (PFM) during different yoga poses varied when measured by electromyography (EMG) in healthy women without urinary symptoms; perianal sensors were used to monitor levator ani activation while four different poses were held for 30 s (locust; modified side plank; side angle; hands-clasped front plank). Activation was highest in the locust pose and least with the front and side planks; the authors concluded that the level of activation in locust was sufficient for strength gains, and that the other poses would improve endurance and/or neuromuscular control [16]. Consequently, yoga combined with engagement of the PFM does appear to be a potentially valuable form of therapy based on the positive results achieved and the level of symptomatic relief reported. Hence the calls for further research to determine the specific mechanisms through which yoga generates a treatment effect, and to identify where yoga has specific benefits [11,12,15].

Fielding et al. first identified the potential of vertically open configuration magnet systems for imaging the PFM in women, including for the evaluation of SUI [17]. Previously the multiplanar scanning capability of MRI and its superior soft tissue differentiation and excellent contrast resolution had made this the definitive modality for diagnosis of urethral and periurethral pathology in women, but imaging remained challenging due to the functional anatomy and behavior of these structures [18]. Upright open MRI studies have since added valuable insights into how dysfunction and displacement of these and other anatomic structures that maintain continence occur, especially in instances where an increase in symptom occurrence and/or severity follows a change in posture [2,3,4,19].

This applies particularly to the effect of gravity on the pelvic floor, as this group of muscles is central to the maintenance of continence as the PFM provides essential support for the pelvic organs. In the context of yoga therapy for UI, it has also been proposed that a beneficial effect on PFM function and strength of the body core may underlie the benefits derived.

Importantly, the bladder neck and urethra are also integral to the maintenance of continence, with support of the mid-urethra thought to be an essential element of urinary continence in women [20]. Consequently, the functional anatomy of this region has been studied extensively, but principally through the use of ultrasound (US) [21,22,23,24]. What is known is that the bladder neck is 2 to 3 cm long; its sub-mucosa contains elastic tissue, and the internal urethral sphincter is located between the neck of the bladder and the upper end of the urethra. The urethra is 2.5 to 4 cm long and 6 mm in diameter in women; it extends forwards and downwards through the PFM behind the symphysis pubis and opens at the external urethral meatus. The urethral sphincter, a layer of circular skeletal muscle fibers, is located in the urogenital diaphragm which suspends the urethra anteriorly from the pubic bone. Dynamic MRI of the PFM in an upright sitting position has established mean values for inward lift and downward movement of the PFM during straining, and shown PFM contraction to be concentric and to move the coccyx in a ventral, cranial direction [2].

Perineal ultrasound (US) has shown that in nulliparous women contraction of the PFM stabilizes the bladder neck [23], and trans-labial US in women with UI, and prolapse can quantify descent of the urethra and bladder outlet against the inferoposterior margin of the symphysis pubis [21,22,25]. Although functional measures obtained with US show that descent of the bladder neck has the strongest association with SUI, and displacement of 25 mm or more is defined as abnormal [26], data on epidemiological determinants of mobility of the bladder neck are scarce [27].

As neither US nor MRI have been used to determine the effects of yoga postures used in the therapy of UI on the position of pelvic structures related to continence, the aim of this pilot study was to investigate this by building on our prior experience using upright open MRI to study the morphology of UI [3,4]. The primary objective was to test the hypothesis that a scanning protocol able to provide imaging with sufficient anatomic detail to visualize the bladder neck and urethra is feasible. The secondary objective was to use the images to analyze yoga poses commonly used in therapeutic regimens for UI where alterations in posture and the effect of gravity influence the pelvic organs, and to identify if changes in the position or morphology of the bladder neck or urethra occur that are potentially relevant to the maintenance of continence.

## 2. Materials and Methods

### 2.1. Subject

An asymptomatic, 41-year-old, healthy woman familiar with the practice of yoga and able to reproduce and sustain specific poses used in therapeutic yoga regimens for UI while in the scanner, and who, as a staff member in research at the university consented to volunteer, being aware that innovative pilot studies were underway using open MRI to explore in fields outside orthopedics.

### 2.2. Upright Open MRI

**Figure 1:**
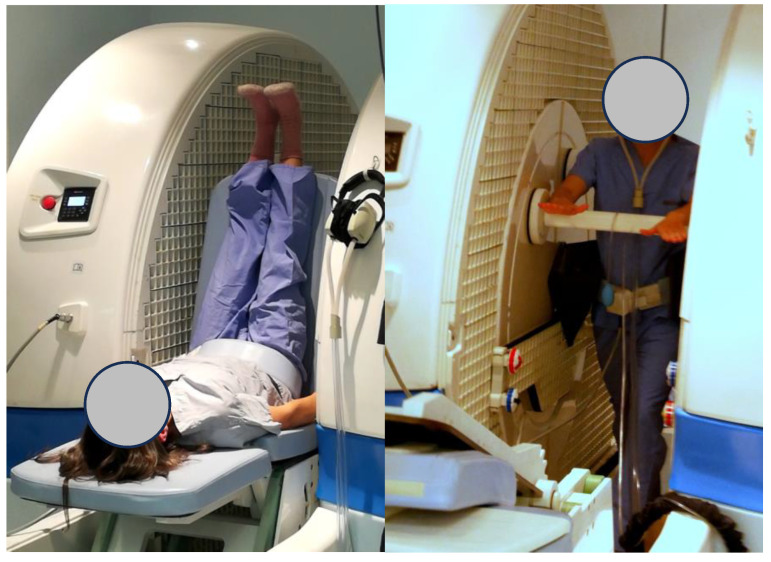
Open magnet configuration of the scanner. These two illustrations show the subject positioned for imaging when standing upright (**right**) and adopting an inversion pose ‘legs up the wall’ (**left**) to demonstrate the capability of the scanner to accommodate changes in posture required in the context of imaging yoga poses. Both images show a one channel surface coil positioned around the pelvis and aids to provide support within the scanner—a foam wedge behind the legs (**left**) and a support bar for the arms (**right**). These aids provide stability and make it easier for the subject to maintain certain poses without movement for the duration of the scan sequence.

Upright open MRI scans of the pelvis were performed using a 0.5 Tesla MRO Open Evo scanner (ASG Superconductors spa, Genova, Italy) located at the University of British Columbia Centre for Hip Health. The configuration of the open MRI scanner with the vertical gap in the magnet is shown in Figure 1; this also illustrates the ability of the scanner to image the pelvis with the subject adopting postures where the effects of gravity on the pelvic organs vary and range from an upright stance to the inversion pose illustrated (this asana was not studied).

### 2.3. Image Acquisition

We used an iterative design, first acquiring supine images to use as a baseline for comparison with images we then obtained of the bladder neck and urethra in yoga poses used in randomized trials of yoga therapy for the treatment of UI [8,9,10]. The poses (asanas) studied were standing Warrior 2 (Virabhadrasana II), supine bridge (Setu Bandha Sarvangasana), and squat (Malasana); images showing these asanas can be viewed online. The volunteer was asked to adopt and hold each pose without additional instruction regarding contracting her pelvic floor.

Because the bladder neck and full length of the course of the urethra were the anatomic regions of interest, we explored imaging using an oblique orientation to capture as much of the urethral lumen along its course as possible. Importantly in terms of image quality, measures were taken which included changing the floor height of the scanner to center the volunteer’s pubic symphysis, and, in addition, for poses where it was possible to do so, supports were used to provide stability to minimize movement artefact.

In each of the yoga poses studied a one-channel surface coil was positioned around the pelvis; for the supine bridge pose, this was a small flex coil aligned with the urethra, and for the squat pose, a large flex coil as the volunteer had to squat into a coil placed around both thighs. The imaging protocol then began by obtaining scout images; these were followed by a series of scan sequences to obtain resting anatomic images of the pelvis where the bladder neck and urethra were visualized. Comparison of the images from the different yoga poses was then made with standing, seated, and supine images from prior phases of our open MRI study [3,4]. This allowed variations in the anatomy of the urethra and bladder neck resulting from the positional effect of each pose to be identified.

## 3. Results

Images with sufficient anatomic detail were obtained in each of the four poses studied to clearly define the bladder neck and urethra.

Optimal imaging was achieved by adjustment of the floor of the scanner to position the pelvis centrally within the field and by the use of physical aids to help the subject to maintain her stability throughout the duration of the scanning sequence.

The sequencing required to obtain the best images in each posture varied due to the iterative, exploratory approach necessary.

Sample images for selected yoga postures that illustrate where the anatomy of the bladder neck and urethra are affected are shown below, accompanied by details of the sequencing used to obtain each series of images from which the examples were taken. These images were chosen to illustrate the ability of open MRI to outline key regions of urethral and bladder neck anatomy relevant to urinary continence.

### 3.1. Scout Images

**Figure 2:**
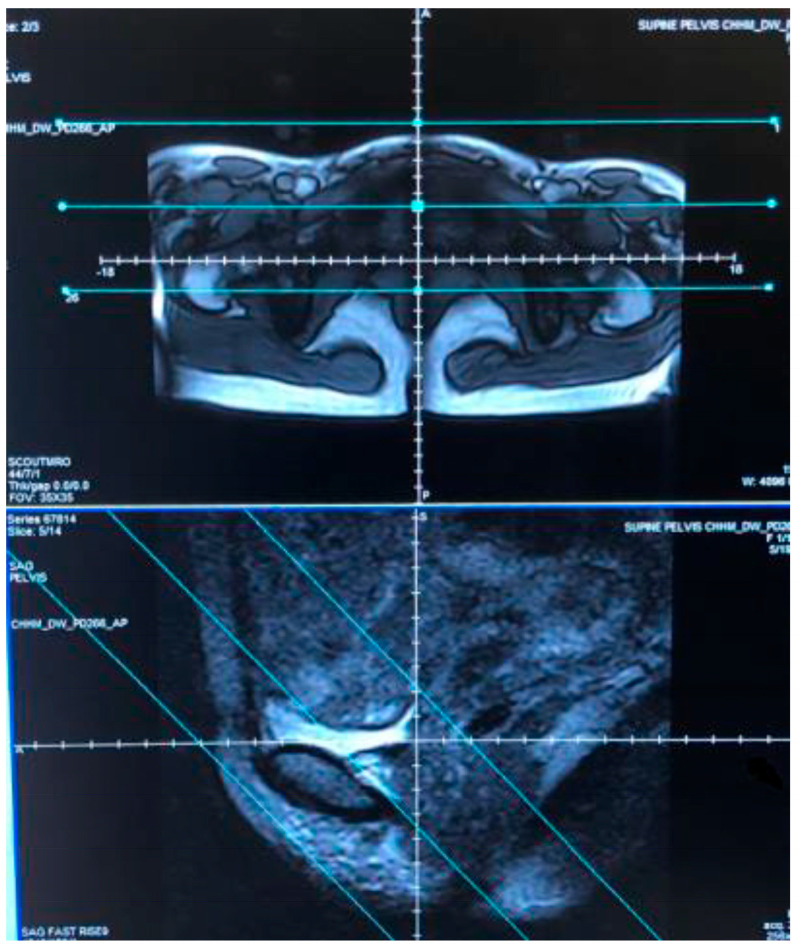
Scout images: The two scans show examples of axial (**upper**) and sagittal (**lower**) scout images which were the first element of each scan sequence. The blue lines indicate the field of view used to ensure the entire bladder and full length of the urethra are included in the protocol.

The first step in the imaging of each yoga posture was to take scout images to ensure that the anatomic region of interest within the pelvis where the bladder and the course of the urethra are centered was within the field of the scanner (Figure 2).

### 3.2. Supine Pose

The sequencing for these images was as follows: T2 weighted Fast Spin Echo (FSE), TR/TE = 4616.8/114 ms, acquisition matrix 192 × 160, NEX = 2, FOV = 30, slice thickness = 2 mm, gap = 1 mm, 16 slices, scan plane—AX, imaging time 5′39″.

**Figure 3:**
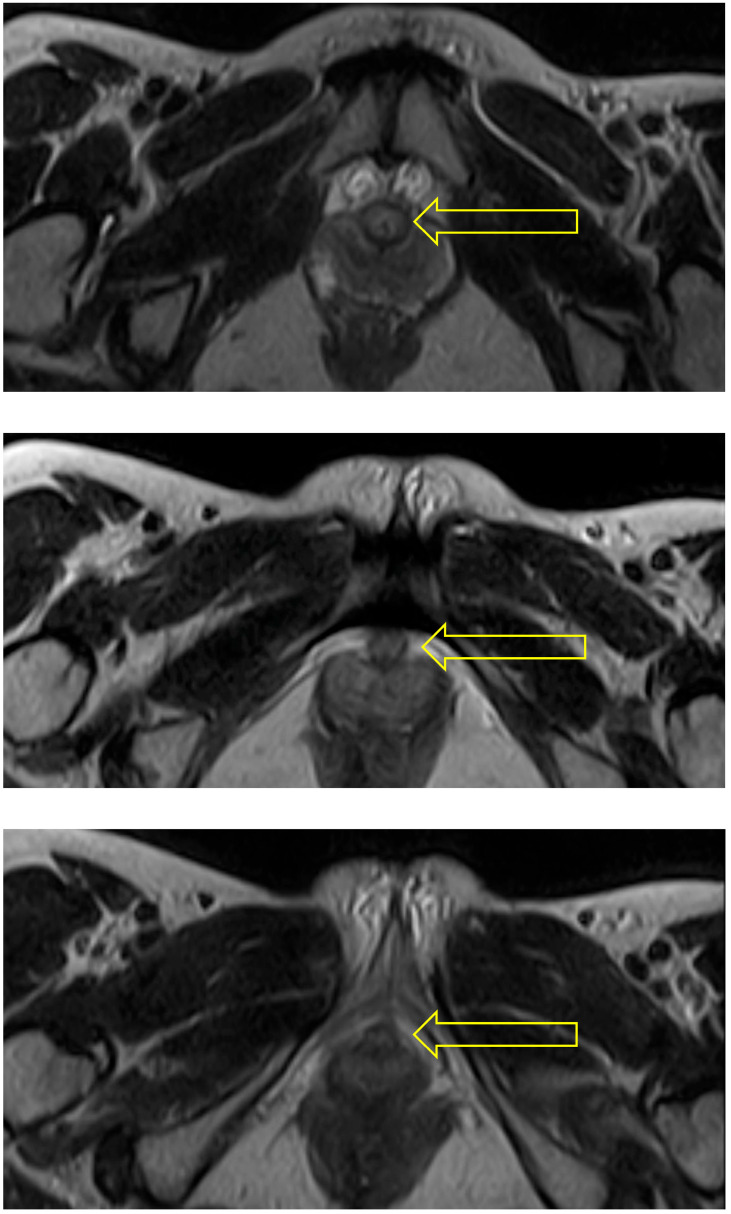
Supine: The 3 scans illustrating this sequence all show the urethra (indicated by arrow) as an uncompressed circular structure at the bladder neck (**top**), distal urethra (**middle**), and towards the urethropelvic ligament (**bottom**).

In the supine pose, Savasana, the urethra is seen throughout its length as a circular structure (see Figure 3).

### 3.3. Squat Pose

The sequencing for these images was as follows: T2 weighted Fast Spin ECHO (FSE), TR/TRE = 4620.8/114 ms acquisition matrix 256 × 192, NEX = 1, FOV = 25 cm, slice thickness = 4 mm, gap = 1 mm, 16 slices, scan plane—AX, imaging time 6′44″.

**Figure 4:**
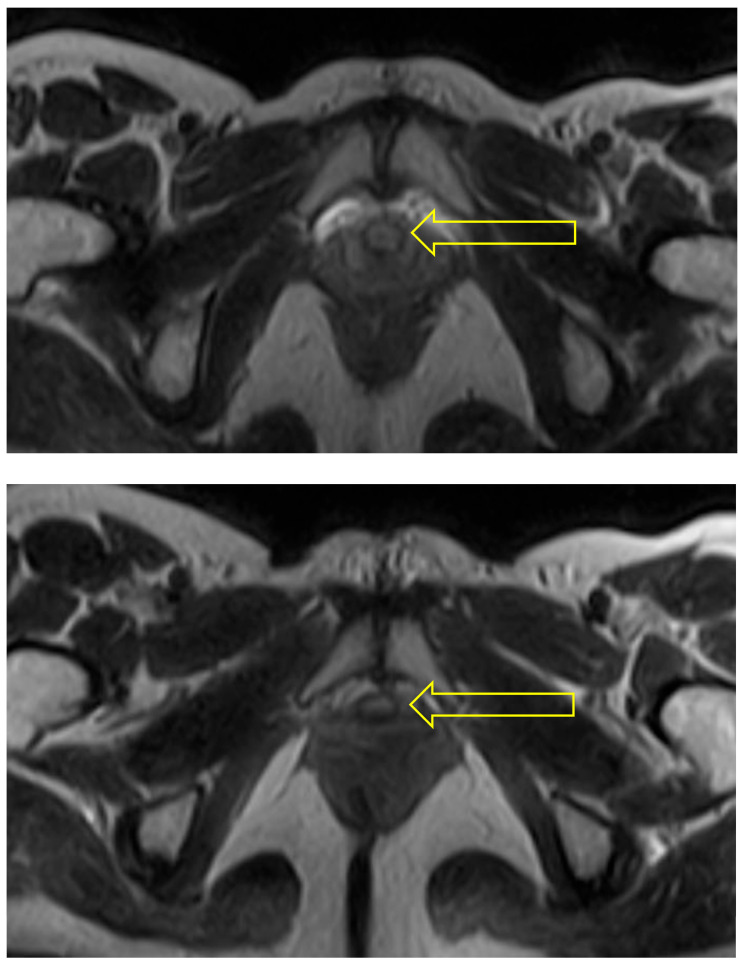
Squat: The two scans selected from this sequence show the urethra (indicated by arrow) as a circular (uncompressed) structure at the bladder neck (**upper**) and oval in shape and hence probably compressed at mid-urethra (**lower**).

In the squat pose, Malasana, the urethra was seen as a circular structure at the bladder neck, but to be become oval in shape in the region of the mid-urethra (see Figure 4).

### 3.4. Supine Bridge Pose

The sequencing for the images below was as follows: T2 weighted Fast Spin Echo (FSE), TR/TRE = 3176/114 ms, acquisition matrix 256 × 192, NEX = 1, FOV = 30 cm, slice thickness = 3 mm, gap = 1 mm, 11 slices, scan plane—AX, imaging time 4′45″.

**Figure 5:**
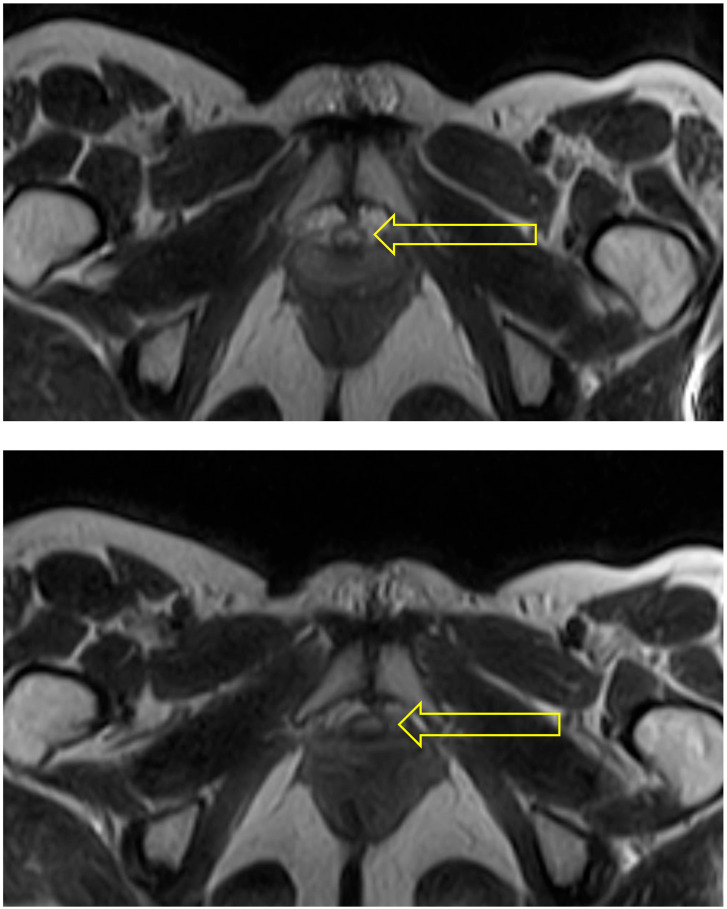
Supine bridge pose: These axial images show an oval shape of the urethra (indicated by arrow) beginning just below the bladder neck (**upper**) and continuing to be evident at the mid-urethra (**lower**). The mid-urethra is known to have a smaller diameter than the distal urethra. The area of hyperintensity around the urethra in comparison with resting state images may indicate pooling of venous blood due to activity within the pelvic floor musculature generated when holding this pose.

**Figure 6:**
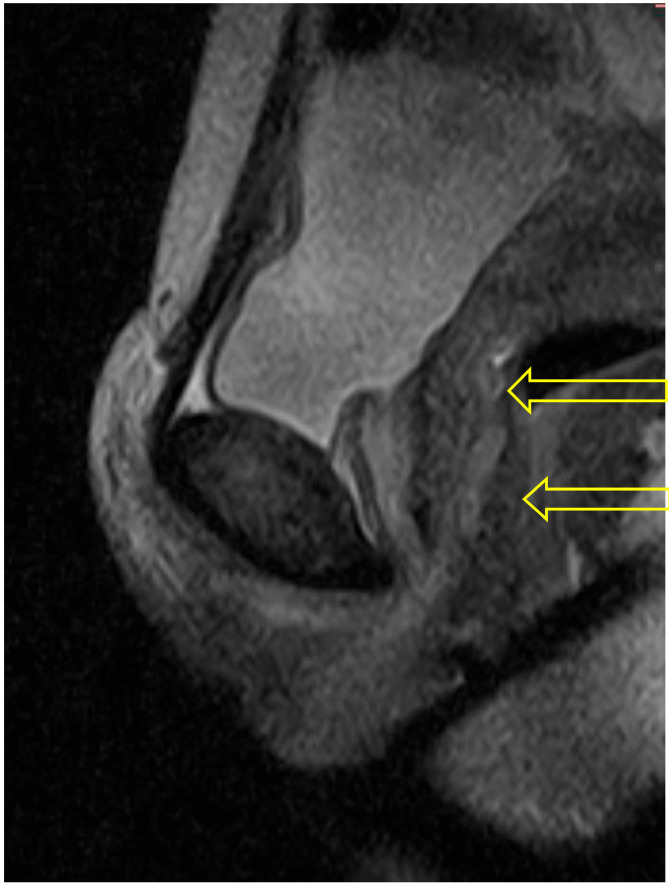
Supine bridge pose: This sagittal scan illustrates a view where the entirety of the course of the urethra can be visualized, and a narrowing is evident at the mid-urethra (lower arrow). The bladder neck is identified by the upper arrow and did not change in diameter or volume from supine to bridge pose.

**Figure 7:**
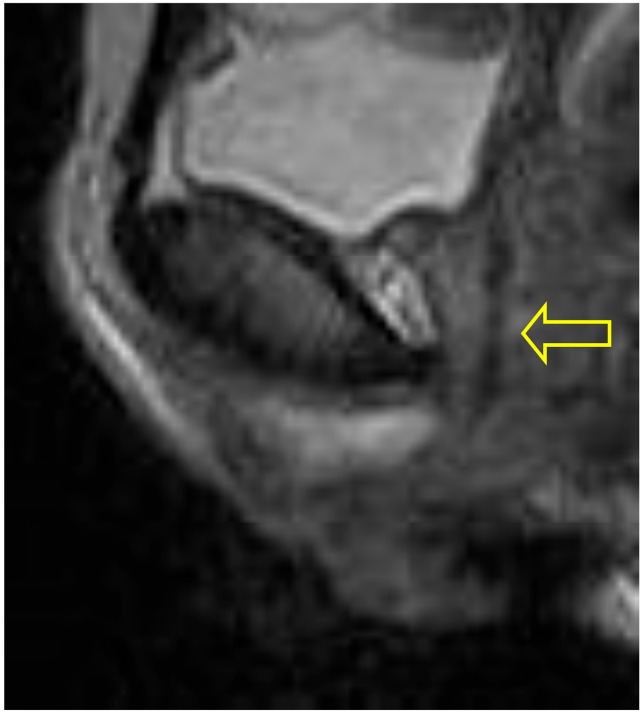
Supine scan: This resting supine scan provides comparison with the supine bridge pose scan shown in Figure 6. In supine imaging the anatomic outline of the urethra (indicated by arrow) shows no regional narrowing.

In the supine bridge pose (Setu Bandha Sarvangasana), the axial images show an oval shape of the urethra beginning just below the bladder neck which continues to be evident at the mid-urethra (see Figure 5). In addition, narrowing was evident in the mid-urethra in sagittal scans (see Figure 6); when measurements were compared to supine baseline images where the mid-urethra measured 12 mm, the supine bridge pose measurement was 8 mm. Comparison with standing posture images also suggested an increase in urethral length during the supine bridge pose, with a change from a baseline of 36 mm to 45 mm (Figure 7).

### 3.5. Warrior 2 Pose

The sequencing for this posse was as follows: T2 weighted Fast Spin Echo (FSE), TR/TRE = 2599/114 ms, acquisition matrix 256 × 192, NEX = 1, FOV = 25 cm, slice thickness = 3 mm, gap = 1 mm, 9 slices, scan plane—SAG, imaging time 3′58″.

**Figure 8:**
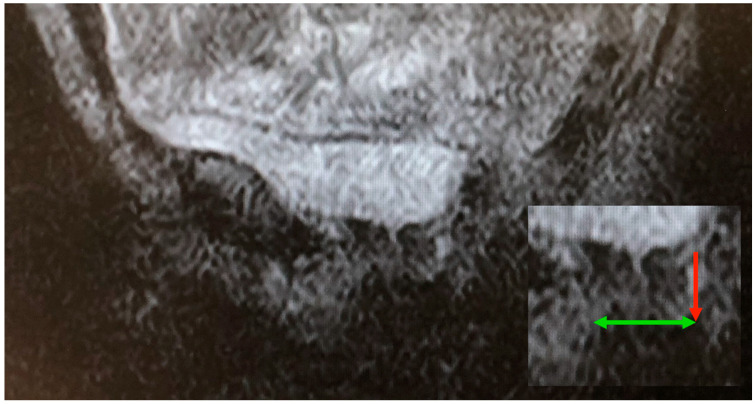
Warrior 2 pose: This scan illustrates the less well-defined image quality obtained during poses where stability cannot be aided through support to minimize movement during a scan sequence of several minutes. However, the bladder neck and urethra are still sufficiently defined to allow for anatomical measurements to be made of lengthening (vertical arrow) and narrowing (horizontal arrow) in insert bottom right.

The Warrior 2 pose (Virabhadrasana II) is one where the subject may have difficulty maintaining stability during the scanning sequence. In this pose, the upright posture, wide stance, extended arm position, and angle and position of the pelvis render the pelvis particularly prone to movement, and these features of this pose combined with the constraints of being in the scanner prevent any of the conventional support mechanisms available in the open MRI environment being used. Hence movement artefact can affect the quality of the imaging (see Figure 8).

However, even in less well-defined scans the key anatomical structures of the bladder neck and urethra can still be visualized, including the oval shape of the urethra, and measurements can be made that are able to quantify the effects of posture and gravity on the caliber and length of the urethra.

### 3.6. Observed Anatomic Effects

Changes in the urethral width, length, and overall structural shape were evident when comparing supine to upright posture. The upright pose (Warrior 2), which is adopted with leg abduction, generated the highest degree of urethral widening, changing from a supine baseline of 12 mm to 21 mm; this change was also accompanied by foreshortening of the urethra, from a supine baseline of 36 mm to 25 mm. In contrast, the supine bridge pose generated changes suggesting this position uniquely narrowed the mid-urethra, and associated measurements also suggested an increase in length from a standing base line of 36 mm to 45 mm. Where the urethral outline became oval in shape, this is presumed to be due to compression generated by effects of the pose on the PFM. Slight funneling of the bladder neck was also evident with the Warrior 2 pose as compared to the supine bridge pose.

## 4. Discussion

This pilot study identified that open MRI can generate scans with sufficient anatomic detail to clearly define the bladder neck and urethra in a healthy woman during yoga poses used in therapy for UI. It builds on previous research comparing scans of women when supine and upright which demonstrated that changes in the urethra and bladder neck occur related to variations in posture which likely reflect the effects of gravity on the position of the pelvic organs [3,4,28].

We suggest that the current study indicates that open MRI has the capacity to help identify the unelucidated changes in pelvic structures believed to underlie the significant clinical benefits shown in randomized controlled trials of therapeutic yoga regimens for women with UI [5,15]. Further studies using open MRI could also answer the call to define which yoga poses achieve an anatomic effect, such as changes in the position and nature (thickness or length) of the bladder neck and urethra. As identified by other authors, it is poses having such an effect that are most likely to help women with UI achieve symptomatic relief [11,12].

This study compliments prior research from when MRI originally became the definitive imaging modality for diagnosing elements of urethral morphology and behavior important in SUI [20,29]. It also adds to other open MRI studies that demonstrated how the function and integrity of the pelvic floor can be influenced by the effects of gravity in an upright posture [2,17,19,28].

The effects of gravity likely relate to the tendency of the abdominal organs to descend when in an upright posture; combined with increased abdominal pressure this can cause significant descent of the bladder neck and rotation of the urethra in women with laxity of the PFM [30,31]. Gravity and abdominal pressure can also overload the PFM and influence the ability to perform satisfactory muscle contractions; [32] beneficial effects of yoga therapy in SUI are likely mediated through improvements in PFM function.

Our findings suggest that yoga poses exert a temporary effect on the position, length or caliber of the urethra, and the thickness of the bladder neck. In the supine pose, Savasana, the urethra was seen throughout its length as a circular structure which we hypothesize is indicative of a neutral resting state without compression from the pelvic floor muscles. In contrast, in the squat pose, Malasana, while the urethra was seen as a circular structure at the bladder neck, it became oval in shape in the region of the mid-urethra which we suggest may reflect compression due to an anatomic effect from the pelvic floor musculature, although the urethral sphincters likely play a role in compressing the mid-urethra as well. A similar finding was observed in the supine bridge pose (Setu Bandha Sarvangasana) and in the Warrior 2 pose (Virabhadrasana II). In supine bridge the urethra was narrowed from just below the bladder neck to the mid-urethra and in additon the urethral length appeared increased when compared to standing posture images. In the Warrior 2 pose, slight funneling of the bladder neck was also evident.

We suggest the effects observed are relevant as it is known clinically that changes in posture significantly affect the ability of many patients to sustain continence. Also, in continent women, anatomic support of the urethra is provided by the mid-urethra, and during PFM contraction continent women can elevate their mid-urethra significantly higher than those with incontinence [20]. Consequently, in women with SUI it could be that factors affecting support of the urethra and altering the width of the bladder neck are such that the functional integrity of these structures and the internal and external sphincters they contain is compromised.

In women with UI who derive a beneficial effect on their symptoms from yoga therapy, immediate temporary effects generated through a yoga regimen may translate over time into beneficial long term anatomic change due to urethral sphincter hypertrophy or an improvement in the pubococcygeal angle and support of the bladder neck [33,34]. In parallel, with a positive functional effect on structures such as the bladder neck and urethra, PFM coordination, activation, and endurance are now known to be important [34,35,36], and where yoga therapy proves beneficial, this likely also follows an improvement in core strength and PFM tone and function [8,16,37]. PFM contraction exercise for example has been shown to be more effective if performed when standing with external rotation of the hip [38]. Improved PFM function could also perhaps result from an additional effect of moving between postures, or practicing poses that facilitate PFM contraction combined with postures that challenge the continence mechanisms.

In this context, the changes in urethral caliber and length that we documented to occur in the supine bridge position support the opinion of other authors that it is the conduct of specific yoga poses rather than yoga practice in general that is most probably therapeutic. Our findings also support concepts suggested by other authors as to the possible mechanical impact of yoga on the pelvic floor and UI [5,11,12] and extend the prior phases of our study of open MRI using the same scanner to identify changes in the staging of pelvic organ prolapse [2,3,28]—a well-recognized anatomic problem underlying SUI and UI in many women. These studies also incorporated the use of pelvic reference lines and 3D modelling [28]; we suggest that in future open MRI studies of yoga postures, these parameters could be explored as additional defining measures, particularly for bladder neck position.

Also, in the context of future research using open MRI, we identified that the use of supports to aid subject stability does contribute to image quality, particularly by enabling the subject to remain comfortable and retain the pose, and thereby limiting movement artefact during scanning sequences that last several minutes. Examples are a foam wedge as used in our imaging of the supine bridge pose (Setu Bandha Sarvangasana) and a horizontal bar suited to some standing poses. We suggest that the approach we followed of employing support measures, wherever the nature of the pose and constraints of the scanner allowed, contributed to the clarity of the scans obtained (e.g., in the supine bridge pose). This element of our protocol would also be relevant in future comparative studies to help achieve reproducibility of the pose of interest between subjects.

We recognize limitations in what we report. This is a pilot study and only involves imaging in a single healthy subject. More data and studies involving women symptomatic for UI pre- and post-yoga therapy are required to confirm that anatomic changes generated during yoga are associated with a treatment effect from this form of therapy. Despite this, we suggest this pilot study has identified upright open MRI as a modality able to contribute to the call to clarify the underlying cause of the improvement that occurs where yoga benefits women with symptomatic UI [11,12,15] and determined which specific poses used in yoga therapy offer women a beneficial treatment effect [7]. In this context, this study cannot separate out the effects of the muscle activity to hold the pose from the muscle activity required to resist gravity.

Not all poses of interest can be supported by a foam wedge or stabilizing bar within the open magnet, hence the reduction of movement artefact needed to improve image quality during scans of some poses remains a challenge; the Warrior 2 pose is an example.

Also, facilities able to image patients in the way we describe are limited, which with the expense involved will preclude widespread application of the imaging we describe to investigate yoga for UI for the time being. But importantly, open MRI does overcome the recognized limitations of conventional imaging being restricted to supine postures [39,40].

The dynamic nature of pelvic floor pathology has raised a question about the ability of even fast MRI imaging to yield optimal and reproducible results [22]. However, the images achieved using the approach and sequences we have described do confirm the feasibility of imaging the bladder neck and urethra with appropriate definition for diagnostic purposes. We suggest open MRI potentially offers a way to elucidate the anatomic impact of yoga therapy, and that further research, especially imaging of women with SUI, will likely detect that functional effects of potential clinical relevance do occur in at least some of the poses currently used in yoga therapy. In addition to shedding further light on the pathology underlying SUI, there is a clinical need to identify which yoga poses are most likely to enable the many symptomatic women to achieve a beneficial treatment effect.

Importantly, yoga is an accessible and globally available therapy that is increasingly widely used to support health [13,41,42,43]; systematic review and meta-analysis of the frequency of adverse events in randomized controlled trials indicates that yoga is as safe as usual care and exercise [14], while multiple trials indicate that various regimens are able to benefit symptomatic women with UI [5,7,8,9,10,11,12]. Importantly, as yoga is a low-risk intervention compared to surgical therapy, with greater understanding of how and why specific yoga poses are effective, yoga-based therapies have the potential to improve the quality of life of many of the millions of women worldwide living with SUI.

## 5. Conclusions

Open MRI can generate scans with the detail required to identify that anatomical changes involving the bladder neck and urethra occur during yoga poses used in the treatment of UI in women. Further research offers a way to identify which specific yoga poses generate a functional effect on structures influencing continence and hence are most likely to provide symptom relief for women through therapeutic yoga regimens.

## Data Availability

The raw data supporting the conclusions of this article will be made available by the authors on request.

## References

[1] Botchu R., Bharath A., Davies A.M., Butt S., James S.L. (2018). Current concept in upright spinal MRI. Eur. Spine J..

[2] Bø K., Lilleås F., Talseth T., Hedland H. (2001). Dynamic MRI of the pelvic floor muscles in an upright sitting position. Neurourol. Urodyn..

[3] Abdulaziz M., Kavanagh A., Stothers L., Macnab A.J. (2018). Relevance of open magnetic resonance imaging position (sitting and standing) to quantify pelvic organ prolapse in women. Can. Urol. Assoc. J..

[4] Friedman B., Stothers L., Lazare D., Macnab A. (2015). Positional pelvic organ prolapse (POP) evaluation using open, weight-bearing magnetic resonance imaging (MRI). Can. Urol. Assoc. J..

[5] Wieland L.S., Shrestha N., Lassi Z.S., Panda S., Chiaramonte D., Skoetz N. (2019). Yoga for treating urinary incontinence in women. Cochrane Database Syst. Rev..

[6] Nygaard I., Barber M.D., Burgio K.L., Kenton K., Meikle S., Schaffer J., Spino C., Whitehead W.E., Wu J., Brody D.J. (2008). Prevalence of symptomatic pelvic floor disorders in US women. JAMA.

[7] Bethel K. (2019). Yoga for Treating Urinary Incontinence in Women: Commentary on a Cochrane Review. Explore.

[8] Huang A.J., Chesney M., Lisha N., Vittinghoff E., Schembri M., Pawlowsky S., Hsu A., Subak L. (2019). A group-based yoga program for urinary incontinence in ambulatory women: Feasibility, tolerability, and change in incontinence frequency over 3 months in a single-center randomized trial. Am. J. Obs. Gynecol..

[9] Baker J., Costa D., Guarino J.M., Nygaard I. (2014). Comparison of mindfulness-based stress reduction versus yoga on urinary urge incontinence: A randomized pilot study. with 6-month and 1-year follow-up visits. Urogynecology.

[10] Nicosia F.M., Lisha N.E., Chesney M.A., Subak L.L., Plaut T.M., Huang A. (2020). Strategies for evaluating self-efficacy and observed success in the practice of yoga postures for therapeutic indications: Methods from a yoga intervention for urinary incontinence among middle-aged and older women. BMC Complement. Med. Ther..

[11] Wein A.J. (2015). Re: A group-based yoga therapy intervention for urinary incontinence in women: A pilot randomized trial. J. Urol..

[12] Griebling T.L. (2020). Re: A Group-Based Yoga Program for Urinary Incontinence in Ambulatory Women: Feasibility, Tolerability, and Change in Incontinence Frequency over 3 Months in a Single-Center Randomized Trial. J. Urol..

[13] Yoga: What You Need to Know. National Center for Complementary and Integrative Health. Updated August 2023. https://www.nccih.nih.gov/health/yoga-what-you-need-to-know.

[14] Cramer H., Ward L., Saper R., Fishbein D., Dobos G., Lauche R. (2015). The Safety of Yoga: A Systematic Review and Meta-Analysis of Randomized Controlled Trials. Am. J. Epidemiol..

[15] Sha K., Palmer M.H., Yeo S. (2019). Yoga’s biophysiological effects on lower urinary tract symptoms: A scoping review. J. Altern. Complement. Med..

[16] Blagg M., Bolgla L. (2023). The relative activation of pelvic floor muscles during selected yoga poses. Complement. Ther. Clin. Pract..

[17] Fielding J.R., Versi E., Mulkern R.V., Lerner M.H., Griffiths D.J., Jolesz F.A. (1996). MR imaging of the female pelvic floor in the supine and upright positions. J. Magn. Reson. Imaging.

[18] Itani M., Kielar A., Menias C.O., Dighe M.K., Surabhi V., Prasad S.R., O’Malley R., Gangadhar K., Lalwani N. (2016). MRI of female urethra and periurethral pathologies. Int. Urogynecol. J..

[19] Bertschinger K.M., Hetzer F.H., Roos J.E., Treiber K., Marincek B., Hilfiker P.R. (2002). Dynamic MR imaging of the pelvic floor performed with patient sitting in an open-magnet unit versus with patient supine in a closed-magnet unit. Radiology.

[20] Rinne K.M., Kainulainen S., Aukee S., Heinonen S., Nilsson C.G. (2010). Dynamic magnetic resonance imaging of the behavior of the mid-urethra in healthy and stress incontinent women. Obstet. Gynecol. Surv..

[21] Kohorn E.I., Scioscia A.L., Jennty P., Hobbins J.C. (1986). Ultrasound cystourethrography by perineal scanning for the assessment of female stress urinary incontinence. Obstet. Gynecol..

[22] Dietz H.P., Wilson P.D. (1998). Anatomical assessment of the bladder outlet and proximal urethra using ultrasound and videocystourethrography. Int. Urogynecol. J..

[23] Peschers U.M., Vodusek D.B., Fanger G., Schaer G.N., DeLancey J.O., Schuessler B. (2001). Pelvic muscle activity in nulliparous volunteers. Neurourol. Urodyn..

[24] Peschers U.M., Gingelmaier A., Jundt K., Leib B., Dimpfl T. (2001). Evaluation of pelvic floor muscle strength using four different techniques. Int. Urogyn. J..

[25] Schaer G.N., Koechli O.R., Schuessler B., Haller U. (1995). Perineal ultrasound for evaluating the bladder neck in urinary stress incontinence. Obstet. Gynecol..

[26] Naranjo-Ortiz C., Shek K.L., Martin A.J., Dietz H.P. (2016). What is normal bladder neck anatomy?. Int. Urogyn. J..

[27] Horosz E., Pomian A., Zwierzchowska A., Lisik W., Majkusiak W., Tomasik P., Rutkowska B., Skalska J., Siemion M., Banasiuk D. (2020). Epidemiological features of the bladder neck rest position and mobility. J. Clin. Med..

[28] Abdulaziz M., Stothers L., Macnab A. (2018). Methodology for 3D image reconstruction of the female pelvis from upright open MRI (MRO) 2D imaging. Biomed. Spectrosc. Imaging.

[29] Tunn R., Goldammer K., Neymeyer J., Gauruder-Burmester A., Hamm B., Beyersdorff D. (2006). MRI morphology of the levator ani muscle, endopelvic fascia, and urethra in women with stress urinary incontinence. Eur. J. Obstet. Gynecol. Reprod. Biol..

[30] Fielding J.R., Griffiths D.J., Versi E., Mulkern R.V., Lee M.L., Jolesz F.A. (1998). MR imaging of pelvic floor continence mechanisms in the supine and sitting positions. Am. J. Roentgenol..

[31] Dietz H.P., Clarke B. (2001). The influence of posture on perineal ultrasound imaging parameters. Int. Urogynecol. J..

[32] Gimenez M.M., Fitz F.F., de Azevedo Ferreira L., Bortolini M.A.T., Lordêlo P.V.S., Castro R.A. (2022). Pelvic floor muscle function differs between supine and standing positions in women with stress urinary incontinence: An experimental crossover study. J. Physiother..

[33] Bø K. (2004). Pelvic floor muscle training is effective in treatment of female stress urinary incontinence, but how does it work?. Int. Urogynecol. J..

[34] Falah-Hassani K., Reeves J., Shiri R., Hickling D., McLean L. (2021). The pathophysiology of stress urinary incontinence: A systematic review and meta-analysis. Int. Urogynecol. J..

[35] Madill S.J., Pontbriand-Drolet S., Tang A., Dumoulin C. (2013). Effects of PFM rehabilitation on PFM function and morphology in older women. Neurourol. Urodyn..

[36] Dumoulin C., Morin M., Bø K., Berghmans B., Mørkved S., Van Kampen M. (2015). Pelvic floor dynamometry. Evidence-Based Physical Therapy for the Pelvic Floor: Bridging Science and Clinical Practice.

[37] Bø K., Herbert R.D. (2013). There is not yet strong evidence that exercise regimens other than pelvic floor muscle training can reduce stress urinary incontinence in women: A systematic review. J. Physiother..

[38] Ishihara H., Maeda N., Komiya M., Mizuta R., Oda S., Naito K., Urabe Y. (2023). Investigation of effective standing posture for increasing activity of pelvic floor muscles: A cross-sectional study. J. Phys. Fit. Sports Med..

[39] Patravali N. (2007). Ambulatory urodynamic monitoring: Are we wasting our time?. J. Obstet. Gynaecol..

[40] Raizada V., Mittal R.K. (2008). Pelvic floor anatomy and applied physiology. Gastroenterol. Clin. N. Am..

[41] Kumar S., Prasad S., Balakrishnan B., Muthukumaraswamy K., Ganesan M. (2016). Effects of Isha Hatha Yoga on Core Stability and Standing Balance. Adv. Mind Body Med..

[42] Tenfelde S., Tell D., Garfield L., Mathews H., Janusek L. (2021). Yoga for women with urgency urinary incontinence: A pilot study. Urogynecology.

[43] Kannan P., Hsu W.H., Suen W.T., Chan L.M., Assor A., Ho C.M. (2022). Yoga and Pilates compared to pelvic floor muscle training for urinary incontinence in elderly women: A randomised controlled pilot trial. Complement. Ther. Clin. Pract..

